# Separation of Peptides with Forward Osmosis Biomimetic Membranes

**DOI:** 10.3390/membranes6040046

**Published:** 2016-11-15

**Authors:** Niada Bajraktari, Henrik T. Madsen, Mathias F. Gruber, Sigurd Truelsen, Elzbieta L. Jensen, Henrik Jensen, Claus Hélix-Nielsen

**Affiliations:** 1 Department of Environmental Engineering, Technical university of Denmark, Kongens Lyngby 2800, Denmark; nano.mathias@gmail.com (M.F.G.); sigut@env.dtu.dk (S.T.); 2 Department of Chemistry and Bioscience, Aalborg University, Copenhagen 2450, Denmark; htm@bio.aau.dk; 3 Department of Pharmacy, University of Copenhagen, Copenhagen 2100, Denmark; ellu57@gmail.com (E.L.J.); henrik.jensen@sund.ku.dk (H.J.); 4 Department of Chemical Technology, University of Maribor, Maribor 2000, Slovenia

**Keywords:** forward osmosis, biomimetic, peptides, rejection

## Abstract

Forward osmosis (FO) membranes have gained interest in several disciplines for the rejection and concentration of various molecules. One application area for FO membranes that is becoming increasingly popular is the use of the membranes to concentrate or dilute high value compound solutions such as pharmaceuticals. It is crucial in such settings to control the transport over the membrane to avoid losses of valuable compounds, but little is known about the rejection and transport mechanisms of larger biomolecules with often flexible conformations. In this study, transport of two chemically similar peptides with molecular weight (*M_w_*) of 375 and 692 Da across a thin film composite Aquaporin Inside™ Membrane (AIM) FO membrane was investigated. Despite the relative large size, both peptides were able to permeate the dense active layer of the AIM membrane and the transport mechanism was determined to be diffusion-based. Interestingly, the membrane permeability increased 3.65 times for the 692 Da peptide (1.39 × 10^−12^ m^2^·s^−1^) compared to the 375 Da peptide (0.38 × 10^−12^ m^2^·s^−1^). This increase thus occurs for an 85% increase in Mw but only for a 34% increase in peptide radius of gyration (*R_g_*) as determined from molecular dynamics (MD) simulations. This suggests that *R_g_* is a strong influencing factor for membrane permeability. Thus, an increased *R_g_* reflects the larger peptide chains ability to sample a larger conformational space when interacting with the nanostructured active layer increasing the likelihood for permeation.

## 1. Introduction

Within the membrane community there has been an increasing focus on the filtration technique forward osmosis (FO), where filtration is driven by a concentration gradient as opposed to the traditional pressure gradient known from reverse osmosis (RO). FO membranes have gained interest in several disciplines and are finding applications in different markets such as seawater and brackish water desalination [1,2], wastewater treatment [3,4,5], treatment of high salinity waters [6,7,8], fertigation [9,10], textile industry [11,12], dairy [13,14], food [15], and beverage [16,17], power generation [18,19], and pharma industry [20,21].

One of the key performance indicators for FO membranes is the rejection of organic molecules. In drinking water or wastewater treatment, high rejection is important to ensure that micropollutants such as pesticides, pharmaceuticals, and endocrine disruptors do not end up in the final product. In concentration or dilution applications, such as downstream processing of pharmaceuticals and fertigation, it is also important to control the transport of other molecules than water across the membrane to avoid loss of valuable components.

Previous studies have focused on the rejection of relatively small organic molecules (*M_W_* < 365 Da) and with a relatively well defined and rigid structure [22]. Therefore, very little knowledge is available for the rejection of larger, more complex molecules. Based on the reported results in previous studies, very high or complete rejection should be expected when filtrating larger molecules. Typically, rejection has been found to be size dependent with higher rejections for larger molecules [22,23]. Size dependent rejection indicates that steric hindrance is the underlying rejection mechanism during filtration and therefore complete rejection should be possible to obtain. In reality however, rejections are not found to be complete but instead only approximately complete with rejection values in the range of 99%–99.9%. This is due to the diffusion of molecules through the membrane matrix, where molecules first adsorb to the active layer, then diffuse across it until they desorb on the support side of the active layer. Recent findings report that for some membranes, especially dense thin film composite (TFC) membranes, rejection is solely due to diffusion and not steric hindrance [23]. However, at the molecular level, diffusion through the membrane matrix must also be a steric process. If molecules can diffuse through the membrane matrix, they must be able to move in between the polymer chains that constitute the selective layer. Following this logic, there could be an upper limit to the size of molecules that are able to diffuse through the membrane and therefore it is interesting to investigate whether larger molecules with complex structures, such as peptides or proteins, are still able to diffuse through the membrane matrix or if they become entirely retained. Ultimately, this will have a large impact not only on the chosen operating conditions of the system but it will also question the stability and robustness of the fabrication process of biomimetic membranes with essential components in the active layer such as proteins, peptides, polymers, and vesicles.

In this study, the rejection and transport mechanism through the membrane barrier of two chemically similar peptides of different molecular size (375 and 692 Da) were investigated with a biomimetic, high rejection, and dense TFC membrane. In an earlier study, the rejection mechanism for the membrane had been found to be diffusion based, even for small molecules (*M_W_* = 149 Da) and the membrane is as such ideal for the purpose of this study [23]. To investigate whether the peptides could move through the membrane and if the transport was due to diffusion, the membranes were exposed to different concentration gradients of peptides in a specially designed FO cell. Furthermore, being complex molecules with no rigid structures, a radius of gyration for each peptide was obtained through molecular dynamic simulations, which then that radius was related to the peptides permeability through the membrane matrix.

## 2. Theory

### 2.1. FO Filtration

In FO, water transport is driven by an osmotic pressure gradient, Δπ [bar], between the feed and draw solution. For an ideal membrane, the water flux, *J_w_* [L·m^−2^·h^−1^], can be described by Equation (1).

(1) $$J_{w}=L_{p}\Delta \pi$$

where *L_p_* [L·m^−2^·h^−1^·bar^−1^] is the membrane permeability coefficient of the membrane.

For the same membrane, salt will diffuse from the draw to the feed solution as a result of the salt concentration gradient, Δ*C* [g·L^−1^], as described by Equation (2).

(2) $$J_{s}=-B\Delta C$$

where *B* [L·m^−2^·h^−1^] is the salt permeability coefficient and *J_s_* [g·m^−2^·h^−1^] is the salt flux.

### 2.2. Separation Performance

The rejection of the solutes in an FO process is described by Equation (3).

(3) $$R=\left(1-\frac{c_{p}}{c_{f}}\right)100\%$$

where *R* [%] is the rejection of the specific solute, *c_f_* is the average concentration of the solute in the feed during experiments and *c_p_* is the solute concentration in the permeate. The average concentration of the solute in the feed during experiments is described by Equation (4).

(4) $$c_{f}=\frac{c_{0}+c_{end}}{2}$$

where *c_0_* is the initial concentration of the solute at the start of the experiment and *c_end_* is the concentration at the end of the experiment. Any concentration unit can be used.

### 2.3. Modeling of Membrane Performance

In a previous study with the same membrane, the rejection of three small organic molecules (atrazine, BAM, and DEIA; 215.69 Da, 190.03 Da, and 145.55 Da, respectively) was modeled with a solution diffusion based model, while a pore flow model could not be fitted [23]. Since the peptides are larger molecules than the previously investigated pesticides and the hydrodynamic model being based on size exclusion, it was assumed that that a pore-flow model would not be applicable for the peptides. Instead, the rejection mechanism was investigated with the Fickian’ Solution-Diffusion model [24,25].

In the solution-diffusion model, the membrane is viewed as a solid barrier, through which the molecules move by first adsorbing to the surface and then diffusing through the membrane matrix until they reach the other side of the membrane from where they desorb and move into the permeate. The flux through the membrane is described by Equation (5).

(5) $$J_{p}=\frac{P}{l}\Delta C_{p}$$

where $J_{p}$ [mol·m^−2^·s^−1^] is the flux of peptides, l [m] is the membrane thickness, *P* [m^2^·s^−1^] is the permeability and $\Delta C_{p}$ [mol·m^−3^] is the average concentration difference across the membrane.

The permeability coefficient can further be described as a function of the diffusion coefficient of the peptide through the membrane and the sorption coefficient of the peptide to the membrane material as shown in Equation (6).

(6) P=DS

where *D* [m^2^·s^−1^] is the diffusion coefficient for the peptide in the membrane matrix and *S* is the sorption coefficient of the peptide to the membrane material. *S* is dimensionless, since it is the ratio of the peptide activity in solution relative to the membrane surface. In practice, it is sufficient to determine the permeability coefficient when modeling the rejection.

Experimentally, the permeability coefficient for each peptide was determined by measuring the flux of peptides across the membrane at different concentration difference values in the absence of water flux. In the assays, the water flux was eliminated by removing the osmotic gradient over the membrane, which in practice was done by using a solution of the same osmolarity on both sides of the membrane. The peptide flux was then determined by measuring the peptide concentration in the permeate after a given amount of time, and the permeability coefficient could be found as the slope of the linear plot.

## 3. Experimental

### 3.1. Forward Osmosis Membranes

Biomimetic FO membranes from Aquaporin A/S were used to study the diffusion mechanisms through the membrane. The membranes contain functional aquaporin channels embedded in vesicles of 200 nm size which are further incorporated in the membrane through a thin film composite active layer formation [26]. The TFC layer is synthetized on a porous support for mechanical strength during the operating procedure.

### 3.2. Selected Peptides

**Figure 1:**
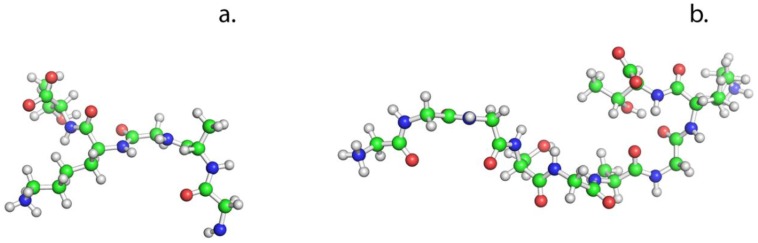
Chemical representation of the two peptides. (**a**) AGKT (375 Da) and (**b**) GGG SGA GKT (692 Da). The peptides are constructs based on the amino acid configuration described by Bianchi et al. [28]. The peptide amine (N–H) backbone groups form a ‘nest’ structure toward phosphate anions and the Lysine side chain participates in the binding when phosphorous compounds are present. Color code: green = C, blue = N, red = O, and white = H.

Two different peptides were used for the up-concentration assay and transport mechanisms experiments, one larger peptide (GGG SGA GKT) of 692 Da and a second smaller peptide (AGKT) of 375 Da, see Figure 1. The two peptides have been tested with both experimental and computational methods. The peptides are expected to be very flexible due to the lack of a rigid secondary structure, making their geometrical size difficult to determine. Thus, the radius of gyration is used as a statistical measure for the size of the peptides, a method that is used to represent the size of macromolecules [27]. Using Molecular Dynamics (MD), see Computational methods section, the radius of gyration is calculated based on the most often occurring conformations. The radius of gyration is then correlated to the permeability of the peptide through the membrane matrix obtained in experimental assays.

From a practical point of view, the two peptides are interesting since they potentially can be used to capture and recover phosphate. The peptide sequence Ser-Gly-Ala-Gly-Lys-Thr (SGA GKT) is a reconstructed P-loop in which proteins are able to bind phosphate groups of GTP and ATP. This hexapeptide has previously been reported to bind to ${\mathrm{HPO}}_{4}^{2-}$ at high pH [28] and may therefore be able to bind phosphorous compounds as a means to address the future issues with diminishing phosphorous resources.

### 3.3. Forward Osmosis Operating System

**Figure 2:**
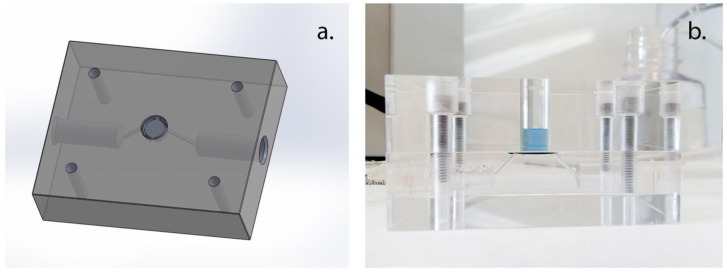
Forward osmosis operating module with static top compartment. (**a**) Showing the drawings of the lower unit where the peptide sample was recirculated and (**b**) showing the assembled module. The two units are separated by a flat sheet membrane tightened with an o-ring and fastened together mechanically through screws.

An FO operating module was designed, built, and used to conduct the experiments, as shown in Figure 2. The device consists of two compartments, a static upper unit containing the buffer solution and a lower unit where the peptide sample was recirculated. The device can also be used for FO experiments, with one of the units containing a draw solution with osmotic potential for up-concentration or dilution of samples. In this study, the device was used to conduct diffusion experiments without the effect of the osmotic gradient, therefore the draw solution was replaced with a buffer solution. Thus, there was only a peptide concentration gradient over the membrane barrier.

### 3.4. Rejection Model Assay

The FO module was used to determine the diffusion of the peptide molecules through the membrane matrix. A vial of each peptide (10 mg) was solubilized in TES buffer (2-[2-Hydroxy-1,1-*bis*(hydroxymethyl)ethyl)amino]ethanesulfonic acid) consisting of 10 mM TES, 1 mM EDTA and the pH was adjusted to 8 with 1 M NaOH to generate stock solutions of 100 mg/L. From this, peptide solutions of 1 mg/L, 5 mg/L, and 10 mg/L were prepared. 0.5 mL of TES buffer (without peptide) was placed in the reservoir compartment and 100 mL of the buffered peptide solution was re-circulated in the bottom compartment. The system was saturated for five hours or overnight to avoid any start up effects such as the loss of peptide to the chamber walls and the membrane. Before starting the diffusion experiment, the reservoir compartment containing the pure TES buffer was emptied and washed with TES buffer. The experiment was initiated by placing a fresh 0.5 mL aliquot of TES buffer in the reservoir compartment and was then set to run for five hours. At the end of the experiment, the TES buffer solution was removed from the reservoir compartment, weighed, and analyzed with HPLC to quantify potential diffusing peptides. The previously described steps were followed for each of the chosen concentrations for both the peptides.

### 3.5. Analytical Methods

The peptides were analyzed with HPLC/UV (1200 Infinity, Agilent Technology, Santa Clara, CA, USA), equipped with a ZORBAX Eclipse XDB-C18 5 µm column (Agilent Technology, Santa Clara, CA, USA). For GGG SGA GKT, an eluent mixture of acetonitrile and MilliQ (Merck Millipore, Darmstadt, Germany) in the ratio 10/90 was used. The injection volume was 50 µL and the flow rate 1 mL/min. For AGKT, the peptides were eluted with a 67 mM phosphate buffer at pH 7.4. The injection volume was 50 µL and the flow rate 0.5 mL/min. The chosen absorption wavelength was 205 nm for both peptides.

### 3.6. Computational Methods

The chosen peptides have a flexible structure making it difficult to determine their geometrical size from a single conformational minima. Thus, the radius of gyration is used as a statistical measure of the geometrical size of the peptides. Molecular Dynamics (MD) was used to determine the radius of gyration of the peptides based on hierarchical clusters from the generated trajectory. Simulations were performed with the molecular modeling packages Amber14 and AmberTools14 [29]. Fully extended peptides were created with LEaP in AmberTools14 using the ff14SB force field parameters [30] and were explicitly solvated using the TIP3P model for water [31] in rectangular boxes with 10.0 Å to each edge from the peptides. The systems were minimized for 10,000 steps: 5000 steps using the steepest decent algorithm and 5000 steps using a conjugated gradient algorithm. The systems were then heated to 300 K over 0.8 ns with a weak constraint of 1 kcal/mol on the peptide (NVT ensemble), and subsequently equilibrated for 0.2 ns without any restraints at 300 K (NPT ensemble). Production simulations were run for 800 ns using the Langevin thermostat with a collision frequency of 1 ps^−1^, and the pressure was set to 1 bar using the Berendsen barostat. Non-bonded interactions were cut off at 10.0 Å, full electrostatics for the periodic system were calculated using the Particle Mesh Ewald approach [32], and hydrogen bonds were restrained using SHAKE [33].

The mass weighted radius of gyration, *R_g_* [Å] from a molecular configuration can be calculated as shown in Equation (7).

(7) $$R_{g}^{2}=\frac{1}{M}\underset{i}{\sum }m_{i}{\left(R_{i}-R_{CM}\right)}^{2}$$

where M is the total molecular mass [Da], $m_{i}$ [Da] is the mass of the *i*^th^ atom with position vector $R_{i}$ [Å]. $R_{CM}$ [Å] is the center of mass and calculated as shown in Equation (8).

(8) $$R_{CM}=\frac{1}{M}\underset{i}{\sum }m_{i}R_{i}$$

To compare the influence of a rigid secondary structure on permeability, the radius of gyration was determined for three pesticides from a previous study [23]. The radius of gyration for both the pesticides and peptides is calculated on all heavy atoms by the use of CPPTRAJ [34].

Table 1 provides an overview of the comparison between the peptides and the smaller pesticides. Here, the permeabilites have been used to calculate a rejection value using Equation (9), owing to the fact that rejection values are more commonly used in membrane studies to indicate the retention of a given compound by the membrane.

(9) $$R=\left(1-\frac{P}{t\times J_{w}}\right)\times 100\%$$

where *t* is the membrane thickness (*m*) and *J_w_* the water flux (m^3^·m^−2^·s^−1^). The equation assumes that feed and retentate concentration are equal, appropriate for low recovery values typically used to determine rejection values and that the concentration difference across the membrane can be approximated to be equal to the feed concentration, which is a good approximation for high rejection values (*R* > 95%).

### 3.7. Data Analysis

To determine the rejection mechanisms, the permeate concentrations from the HPLC measurements were used to determine the molar flux of the peptides. The concentration of the peptide was multiplied with the volume in the draw chamber (0.5 mL) and divided with the membrane area (0.785 cm^2^) to generate the peptide flux in mol·s^−1^·m^−2^. The molar flux was then plotted as a function of the concentration difference across the membrane. A linear plot shows a diffusion driven process where the slope is the permeability coefficient, *P*, from Equation (6).

## 4. Results and Discussion

### 4.1. Peptide Transport Mechanism

**Figure 3:**
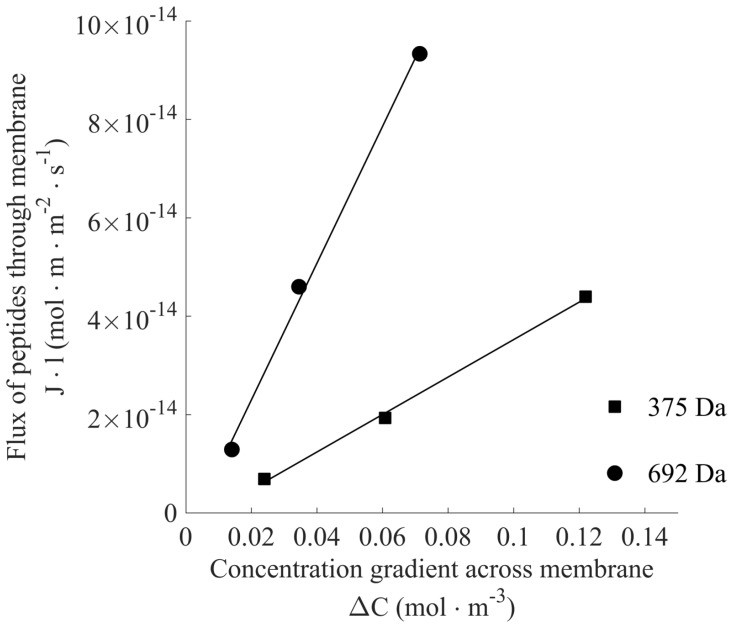
Molar flux of peptides across the membrane. The graph shows the molar peptide flux over the membrane as a function of the concentration gradient across the membrane. Each concentration point is a mean value of triplicate experiments at that concentration. The slope of the graph gives the membrane permeability for each peptide. The linear regression coefficients are 0.99792 (375 Da) and 0.99817 (692 Da).

The peptides were found to be able to pass the membrane, although in small amounts. Roughly less than 1% of the peptides were observed to pass the membrane barrier. From the plots in Figure 3 it can be seen that the molar flux of the peptides is linearly correlated to the concentration gradient across the membrane. This strongly indicates that the transport mechanism is based on diffusion as described by the solution-diffusion model.

Interestingly, the larger peptide was found to have a higher permeability. Based on steric considerations, the larger peptide would be expected to show a lower permeability, since its larger size would make it more difficult to diffuse through the membrane matrix. Furthermore, similar constructs of the peptides have previously been described to exhibit flexible structures, where the peptides can assume several different conformations. To further investigate whether the permeability is affected by the flexibility of the peptides, MD simulations were performed.

### 4.2. MD Analysis of Peptide Structure

**Figure 4:**
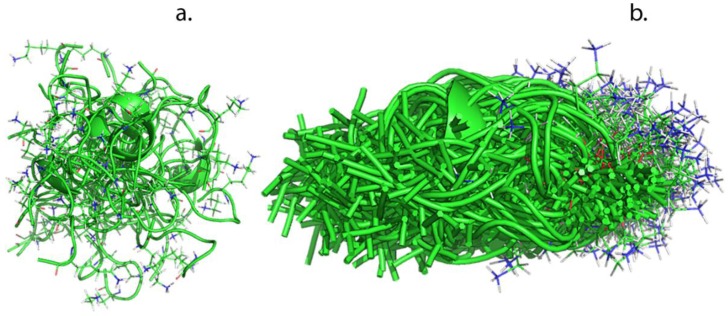
Hierarchical clusters of peptide simulations in water. (**a**) AGKT and (**b**) GGG SGA GKT. The backbone is shown in green and the lysine side chain is marked in stick representation. The illustrations show an ensemble of the same peptides in the many different structural conformations they can exhibit.

Molecular dynamic simulations were run to determine the radius of gyration of each peptide respectively. Hierarchical clusters of the peptides are shown in Figure 4.

From Figure 4, it can be seen that the peptides are shown to exhibit a disordered nature, assuming several different conformations, with the lysine residue in indeterminate orientations. This is in accordance with a previous study where similar constructs (SGA GKT) of the peptides were investigated by MD [35]. Here the peptide of inspiration (SGA GKT) was studied as peptide-only and peptide-phosphate systems. The peptide conformational ensemble was stabilized by the presence of a phosphate anion, however the isolated peptide was likely to spend most of the time in a disordered state [35]. Thus, it is expected that the peptide will exhibit a flexible structure when in contact with the membrane matrix.

From the molecular dynamic simulations, the radii of gyrations are obtained. These are shown in Table 1. The GGG SGA GKT peptide has a radius of gyration 34% larger than the AGKT peptide. The main difference between the two peptides is the additional GGGS amino acids on the larger peptide, which primarily makes this peptide longer and not wider, since these amino acids have short side chains. The longer chain provides the GGG SGA GKT peptide with a larger number of degrees of freedom for conformational operation and for these two peptides the higher radius of gyration of the larger peptide is as such also correlated to a greater flexibility and ability to bend, which have been found to play a role in the transport of larger molecules through membranes [36,37].

### 4.3. Effect of Size, Flexibility, and Polarity in Membrane Transport

**Figure 5:**
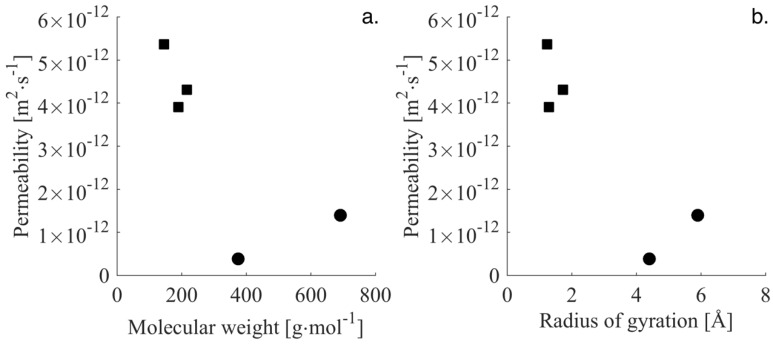
Permeability as a function of molecular weight (**a**) and radius of gyration (**b**). As a comparison, three previously tested pesticides are shown in ■ symbols. The two peptides are shown in ● symbols.

Size is still an important descriptor for permeability as shown in Figure 5a. Here the permeabilities of the two peptides are compared to those determined previously for the smaller pesticides (DEIA, BAM, and Atrazine) and this plot clearly indicates that there is a general tendency for larger molecules to show lower permeabilities. The permeability of the pesticides is 5 to 10 times higher than for the peptides. In Figure 5a, molecular weight is used to represent the size of the molecules, but molecular weight might not be the best descriptor of size since it fails to take the complexity of the peptide backbone chains into account. Compared to smaller molecules, it is not easy to fit large molecules with a simple geometric shape such as a rectangle or a cylinder. To better accommodate the geometric structure of the peptides, permeabilities have been plotted as a function of radius of gyration in Figure 5b and it can be seen that this offers a better description. As argued before, for these peptides, the radius of gyration is correlated to the degree of freedom and this may be important for large molecules to pass the membrane barrier. The hypothesis is based on the increase in entropy with an increasing number of conformations, which in practice means that the larger peptide can twist and bend its way through the membrane layers and ultimately be traced on the draw side.

Another factor influencing the permeability of the peptide through the membrane is their polarity and charge. Polarity can influence the sorption of the peptides to the membrane surface, which would be the first step in a solution-diffusion based transport mechanism, and the orientation of the molecule towards the membrane surface [38]. Charge may also influence peptide orientation towards the membrane surface and may lead to either attraction or repulsion depending on the relative charge between peptide and membrane. Bianchi et al. investigated the charge of the SGA GKT peptide as a function of pH and, based on these results, half the population of AGKT peptides are expected to carry a positive charge in these experiments. The same is estimated for the GGG SGA GKT peptide. The additional amino acids in the peptide structure carry no charge-bearing side groups and the N-terminal of alanine and glycine have very similar pK_a_ values (9.69 and 9.60) [39]. The charge of the membrane has not been determined in this study. However, the membrane is a TFC membrane and these membranes typically have a negative surface charge. Based on this and a previous study where the AQP membrane was found to interact with cationic flocculants, the AQP membrane is most likely negatively charged. The opposite charge of the membrane surface and the peptides will allow for attraction. The peptides can as such become ionically bound to the negatively charged carboxylic acid surface groups and the diffusion through the membrane matrix could then occur with the peptide jumping from one negatively charged surface group to the next. This might also be used to explain why the larger peptide has the highest permeability. Compared to the smaller peptide, it has an additional chain of four amino acids (GGG S), and this may make it more difficult for the positively charged side group of the larger peptide to get into contact with the negative surface groups. It would interact less with the membrane surface and its diffusion through the membrane matrix would be relatively free. In comparison, the AGKT peptide would be slowed down by the negative surface groups and thus experience a lower permeability.

In Table 1, the 95% confidence interval for the peptide permeability coefficients are also given. Compared to the permeability coefficients, they are relatively large, and this reflects the heterogeneity of the membrane surface. The permeability coefficients were determined for several incremental pieces of the same membrane sheet, and were found to vary significantly between pieces. The confidence intervals can thus be understood as a measure of the heterogeneousness of the membrane surface.

The finding, that a molecule like the 692 Da peptide is capable of penetrating a selective layer that has been found to show almost complete rejection of molecules that are 4.5 times smaller (DEIA, 146 Da) is highly interesting. It shows that for even dense and highly rejecting membranes, permeation of significantly larger molecules is still possible. This phenomenon in FO filtration might lead to a slow accumulation of the molecules in the draw solution, especially if the draw solution is recovered with membrane distillation and the molecules have a low volatility, which is the standard for biomolecules. The membrane penetration of the compounds might also ultimately alter the membrane performance. This is especially true for compounds that have a higher affinity for the membrane material and as such move into the structure and accumulate, which may ultimately change membrane characteristics. Also, within the FO community there is a constant search for the holy grail of draw solutes. If potential candidates are toxic even in very low concentrations, it is crucial to know that they might cross the membrane barrier to the feed side even when the size of the draw solute is relatively large.

Finally, biomimetic membranes are based on the concept that biologically active molecules are incorporated in the selective layer of the membrane. This study shows that biomolecules not too dissimilar to proteins are capable of diffusing through the membrane matrix. The question is then at which size does it become completely impossible for these molecules to diffuse through the membrane. Biomimetic membranes and in general mixed matrix composite membranes are based on the incorporation of particles in the selective layer. The results of this study indicate that ultimately the structure of a mixed matrix membrane might not be static, leading to a leakage of membrane components to the surrounding medium. One way to overcome this could be to covalently bind the proteins in biomimetic structures to their surrounding environment in the selective layer.

## 5. Conclusions

In this study, the transport of two peptides of molecular sizes 375 Da and 692 Da with flexible structures across a biomimetic forward osmosis membrane with a dense selective layer was investigated. The membrane exhibited high but incomplete rejection rates where 1% of the peptides were still able to cross the membrane barrier in spite of their relatively large size. The peptide with the smallest molecular mass was found to have the lowest permeability, which might be explained by the radius of gyration. A higher radius of gyration enables the peptide to assume several conformations when in contact with the membrane matrix, which ultimately could increase the probability of the peptide being transported through the membrane. The transport mechanism was found to be diffusion based. The overall finding of this study was that even relatively large molecules can cross an otherwise dense membrane layer. This is significant primarily for choosing the optimal operating conditions and run time of the system in downstream processing and needs to be considered for each and every forward osmosis application. Ultimately, it has a significance in the robustness of the membrane fabrication process where the incorporation of biomolecules in the selective layer is one of the key steps.

## Figures and Tables

**Table 1:** Overview and comparison of membrane transport parameters. The table shows characterization parameters for each peptide and a comparison to three pesticides in a previous study with the same membrane [23]. The rejection values are calculated on measured permeabilities and a water flux of 9.71 L·m^−2^·h^−1^ reported in the previous study. The membrane thickness was measured to be 112 μm and the active area for the setup was 0.785 cm^2^. The flux is based on a feed concentration of 1 mg·L^−1^.

Compound	Permeability (m^2^·s^−1^)	Molecular Weight (g·mol^−1^)	Radius of Gyration (Å)	Flux (μg·m^−2^·h^−1^)	Rejection (%)
Peptide					
AGKT	0.38 × 10^−12^ ± 1.14 × 10^−12^	375	4.4 ± 0.1	12 ± 37	99.9 ± 0.4
GGG SGA GKT	1.39 × 10^−12^ ± 0.9 × 10^−12^	692	5.9 ± 0.4	45 ± 29	99.5 ± 0.3
Pesticide					
DEIA	5.36 × 10^−12^	146	1.24	172	98.2
BAM	3.9 × 10^−12^	190	1.30	125	98.7
Atrazine	4.31 × 10^−12^	216	1.73	139	98.6
